# The Effects of Commercially-Relevant Disturbances on Sleep Behaviour in Laying Hens

**DOI:** 10.3390/ani13193105

**Published:** 2023-10-05

**Authors:** Endre Putyora, Sarah Brocklehurst, Victoria Sandilands

**Affiliations:** 1 Department of Veterinary and Biosciences, Faculty of Veterinary Medicine, Ghent University, 9820 Merelbeke, Belgium; 2 Department of Agriculture, Horticulture and Engineering Sciences, Scotland’s Rural College (SRUC), Edinburgh EH25 9RG, UK; vicky.sandilands@sruc.ac.uk; 3 Biomathematics & Statistics Scotland (BioSS), Edinburgh EH9 3FD, UK; sarah.brocklehurst@bioss.ac.uk

**Keywords:** welfare, recovery sleep, wakefulness, SWS, REM sleep, EEG, temperature, feed deprivation, bumblefoot

## Abstract

**Simple Summary:**

The welfare of farm animals is important to the animal, farmers and consumers. Sleep is sensitive to disturbances and may be a useful tool in assessing welfare at night. The objective of this study was to look at the effects of 24 h disturbances (feed removal, increased room temperature, footpad pain) on sleep behaviour in laying hens. Sleep during lights-off comprised both slow-wave sleep (SWS) and rapid eye movement (REM) sleep. Averaged over all nights, behaviour during lights-off consisted of 60% SWS and 12% REM sleep, with the remaining 28% attributed to being awake. During lights-off, feed removal and footpad pain had little to no effect on behaviours, while increased room temperature nearly eliminated REM sleep and reduced SWS. During lights-on, footpad pain increased the amount of time hens spent resting and in SWS, with no effects seen for feed removal or increased temperature. Global warming and subsequent increased poultry shed temperatures are likely to result in reduced sleep and welfare in on-farm laying hens, while lack of feed and footpad pain may disrupt sleep less.

**Abstract:**

Ensuring the welfare of commercially kept animals is a legal and ethical responsibility. Sleep behaviour can be sensitive to environmental perturbations and may be useful in assessing welfare state. The objective of this study was to use behavioural and electrophysiological (EEG) measures to observe the effects of 24 h stressors followed by periods of no stressors on laying hen sleep behaviour, and to investigate the use of sleep behaviour as a means of welfare assessment in commercial poultry. Ten laying hens surgically implanted with EEG devices to record their brain activity over four batches were used. Hens were subjected to undisturbed, disturbed and recovery periods for 24 h. Disturbed periods consisted of either feed deprivation, increased ambient temperature (28 °C) or simulated footpad pain via injection of Freund’s adjuvant into the footpad. Sleep state was scored using behaviour data from infrared cameras and EEG data. Over all periods, hens engaged in both SWS (average 60%) and REM sleep (average 12%) during the lights-off period. Feed deprivation and footpad pain had little to no effect on sleep states, while increased ambient temperature significantly reduced REM sleep (to near elimination, *p* < 0.001) and SWS (*p* = 0.017). During the lights-on period, footpad pain increased the proportion of time spent resting (*p* = 0.008) and in SWS (*p* < 0.001), with feed deprivation or increased ambient temperature (*p* > 0.05) having no effect. Increasing ambient temperatures are likely to affect sleep and welfare in commercially-kept laying hens in the face of global climate change.

## 1. Introduction

Maintaining the welfare of commercial animals is both an ethical and legal responsibility. Animal welfare can be defined as an animal’s emotional perception of its environment, how an animal navigates its environment, and how an animal compensates for disruptive changes in its environment by employing physiological and behavioural adjustments [1,2]. To this effect, welfare assessments are required to ensure that suitable environmental conditions are being put into practice [3]. Visual, aural and physiological observations of both animal behaviour and physical condition are the best and most direct ways of assessing welfare in farm animals and producing a more complete picture of an animal’s wellbeing [4,5,6]. Furthermore, the presence or absence of certain behaviours can be used as an indicator of animal welfare [7]. There are many factors that can affect the welfare of poultry, and while many of them are known, such as the effects of stocking density [8] and the provision of suitable enrichments to encourage natural behaviours [9], others are less well understood. One such behaviour that has thus far received little attention with regard to its effects on welfare in domestic chickens is resting and sleep behaviour.

A study by Forslind et al. [10] noted that the frequent disturbance of resting broilers by active individuals may result in reduced welfare of the disturbed individual. Additionally, the inability to sleep sufficiently and at the right times can negatively affect energy expenditure, growth, adaptability, behavioural expression and overall welfare of poultry [11,12,13,14]. Reduced sleep may also affect production by causing less growth, increased illness, and possible death [15,16]. Despite the apparent importance of sleep as a component of welfare, there is often no consideration of sleep behaviour in poultry behaviour and welfare assessments. While this might be due to a lack of use of technology in observing animals at night (though, see [17]), there remains a large gap in our understanding of sleep behaviour and its applications in the assessment of poultry wellbeing.

Sleep is typically defined either electrophysiologically (i.e., using brain activity) or behaviourally. The behavioural definition of sleep follows five key characteristics: (1) an elevated arousal threshold, (2) rapid reversibility from this state with sufficient stimulus, (3) a stereotypic or species-specific sleep posture, (4) behavioural quiescence, and (5) the presence of rebound sleep following a period of sufficient deprivation [18,19]. The electrophysiological definition divides sleep into two main states: rapid eye movement (REM) sleep and slow-wave sleep (SWS), also referred to as non-rapid eye movement (NREM) sleep. REM sleep is distinguished by low-amplitude high-frequency brain waves, while SWS is typified by high-amplitude low-frequency waves [20,21,22]. The electrophysiological definitions of sleep also have specific associated behaviour characteristics. SWS is generally characterized by a reduction in muscle tone and associated behavioural quiescence [23], while REM sleep is identified by rapid eye movements, complete muscle atonia with occasional twitching of the facial muscles and extremities, and irregular heart rate and breathing [18,24]. 

Sleep in birds is remarkably comparable to sleep in mammals: birds engage in both SWS and REM sleep, though exhibit much less REM sleep than that seen in mammals [11,25]. The muscle atonia that is characteristic of mammalian sleep is limited to the neck musculature, and occasionally the wings in birds [26,27,28]. Birds are also able to engage in unihemispheric SWS, wherein one hemisphere and respective contralateral eye are active and open, while the other hemisphere and eye is sleeping and closed, respectively [24,26,29,30,31,32,33]. 

Our previous study [34] looking at sleep behaviour in laying hens established a baseline for the proportion of time that hens spend awake or engaged in either SWS (58%) or REM sleep (18%). It also looked at the effects of short-term disturbances (noise, wind and light) on sleep. We found that the disturbances were effective in disrupting sleep during their application, and that birds compensated for this when disturbances stopped by showing increased sleep and decreased wakefulness during the non-disrupted remainder of the night. Hens did not show any carry-over effects into the following day or night. This finding was likely due to a combination of the duration and intensity of the presented disturbances being brief.

In this study, we investigate what effects longer-term and commercially-relevant disturbances would have on sleep in laying hens, namely food deprivation, heat stress, and foot pain. Feed deprivation is not often thought of as being a problem in modern poultry production; however, it is actively used as a means of inducing moulting in laying hens in some countries (see [35,36]). The negative welfare implications of this practice may be magnified if sleep were also to be impacted. Studies in rodents have shown that feed deprivation results in a decrease in REM sleep that returns upon refeeding [37,38,39]; however, no studies have looked at the same effects of feed deprivation on sleep in poultry. Furthermore, it has been well established that engaging in sleep prevents active thermoregulatory behaviours such as shivering or panting [40,41,42]. Given this link between thermoregulation and sleep, it stands to reason that a sufficient increase in ambient temperature would have a negative effect on sleep behaviour in hens. Lastly, several studies have noted the effects of housing type on the generation of conditions such as bumblefoot (e.g., [43,44,45]), which is an infection and swelling of the footpad that is common in commercially-kept poultry. To date, none have looked at the effects of this potentially painful condition on sleep behaviour. However a study by Palma et al. [46] showed that electroshocks on the feet of rats altered sleep behaviour, and it is known that pain in humans affects sleep quantity and quality (as reviewed in [47]). Based on this evidence, it is likely that pain has an equally detrimental effect on sleep in laying hens. 

The aim of this study was to combine behavioural and electrophysiological (EEG) measures to observe the effects of longer-term (24 h) stressors followed by periods of no stressors on laying hen sleep behaviour, and to provide further evidence for the use of sleep behaviour as a means of welfare assessment in commercial poultry.

## 2. Materials and Methods

### 2.1. Animals

Hens were used in a previous study, as detailed in [34]. In brief, we collected 19 H&N Brown Nick (brown-feathered) laying hens from a commercial flock at 59 weeks of age, and a further 3 hens at 65 weeks of age. Upon arrival, all birds were weighed, wing-tagged (Ketchum wing tags, Putham, UK) and placed in the holding room. After EEG implantation, recovery, and use in an earlier study [34], there were 10 remaining implanted birds (2 experimental birds could not be used post-EEG surgery due to a failure in logging equipment, and so are not referred to further) and 6 companion birds, with an additional companion bird not used in the study. The 10 birds were divided into 3 experimental batches (3 hens per batch), while a further 3 hens were collected from the same commercial flock at 65 weeks of age to make up batch 4 (Table 1). 

### 2.2. Housing

Birds were housed in two different rooms as per the experimental protocol in [34]: one for general holding, and one for experimental treatments after EEG implantation. Both rooms were similar in size, had concrete floors, LED lighting (one light above each pen), and were automatically heated and ventilated to maintain the room at 21 °C. Lights were on daily from 05:00 to 19:00 (14L:10D) to reflect a standard commercial egg production environment. Birds were housed individually in pens (2 × 1 m) in both rooms (with the exception of some companion birds in the holding room that were paired up due to space restrictions) to prevent damage to surgical implants and EEG devices; said pens were made of wooden frames with wire mesh sides (5 × 5 cm) that were 1 m high to ensure birds had visual contact with conspecifics. Further wire mesh (1 × 1 cm) walls extended another 0.5 m to prevent hens escaping. Pen floors contained wood shavings as litter. Birds were provided with ad libitum access to layer pellets (16% crude protein and 11.5 MJ energy; ForFarmers UK Ltd., Rougham, UK) and water from bell drinkers. Each pen also contained approximately 1 kg of alfalfa hay (topped up as required), and a wooden perch with a rounded square shape (length 30 cm, height 40 cm).

### 2.3. Surgical Implantation of EEG

**Figure 1:**
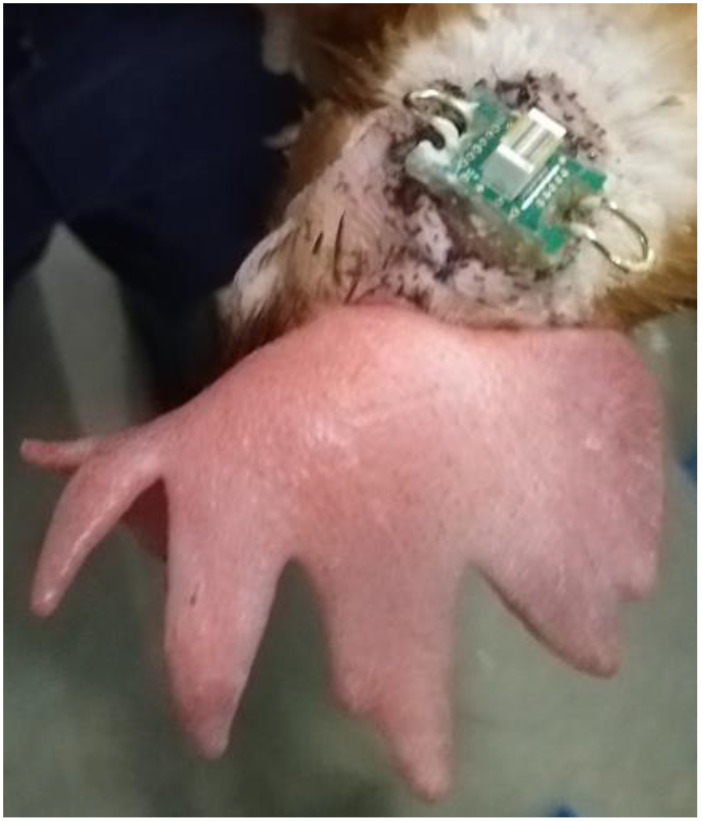
Implanted electronic interface board (EIB) microchip with metal hooks on either side to secure the logging device with rubber bands.

The EEG implants and the procedures for surgical preparation and implantation are identical to those used in [34] (Figure 1). In brief, three birds per day underwent general anaesthesia and EEG implantation at the level of the dura. When birds were fully awake and standing independently, they were brought to the experimental room, where they were housed individually in a row of five hens (companion birds at each end, with three experimental birds in the middle).

### 2.4. Post-Surgical Recovery and Monitoring

Birds were monitored during post-operative recovery for six days (during which time they were also habituated to the experimental pens). This period was followed by the first trial studying the effects of short-term disturbances on sleep (see [34]), after which EEG devices were removed and birds were left undisturbed for seven days.

### 2.5. EEG Devices

EEGs were monitored and recorded using the ONIEROS device (ViewPoint; Lyon, France). The device weighed 56 g (no more than 3.4% of body weight of the lightest bird used) and measured 3 cm × 2 cm × 1.5 cm. Each device was clicked into the surgical implant and was further secured with a rubber band using omega-shaped hooks. Devices were removed at 08:30 every morning during routine husbandry and charged for 1 h (maximum charging time required), after which they were re-attached. This allowed for 23 h of continuous EEG recording for each bird.

### 2.6. Data Recording

**Figure 2:**
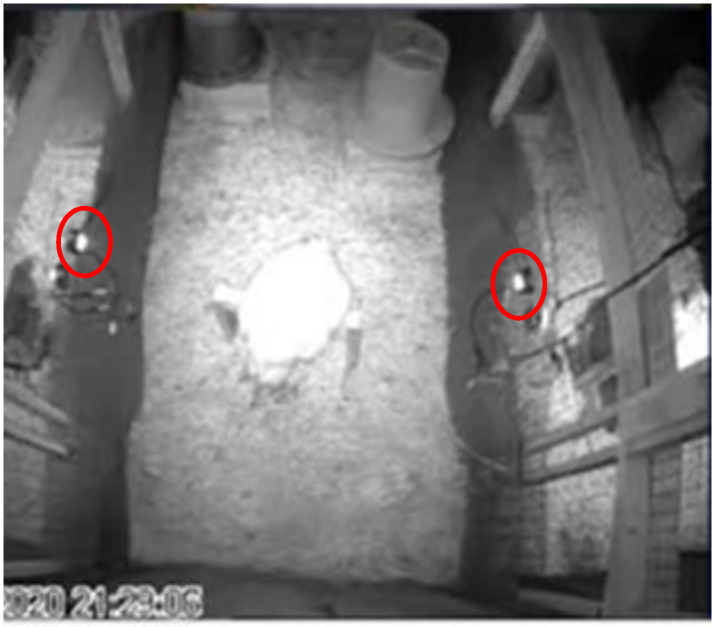
Camera framing displaying an overhead view of the pen with the bird perching for recording during lights-off. The positioning of cameras on either side of the perch is indicated in red.

EEG data were collected for 23 h per day and parcelled as per the methods outlined in [34]. Each experimental pen was outfitted with three infrared cameras: two H.264 CCTV IMX323 night vision IR mini cameras (Ailipu Technology Co., Ltd.; Shenzhen, China) on either side of the perch, and a third high-resolution varifocal camera (Twilight CCTV; Merseyside, UK) pointing downwards from the top of the pen (Figure 2). All cameras were connected to a computer with Geovision NVR Version 8.5 (Taipei, Taiwan) recording software which recorded and saved all video files in ‘.avi’ format. EEG and behavioural recordings were synchronized across devices.

Each experimental batch of hens was recorded using EEG and video cameras for six 23 h periods. Recording periods started at 09:30 (during lights-on) and lasted through the remaining lights-on period, through 10 h of lights-off (19:00–05:00), and until 08:30 the following calendar day. Birds were subjected to one of the following periods in a single day: undisturbed, disturbed or recovery. The three disturbance treatments were (1) feed deprivation (feeders were removed from all pens at 08:30 and returned the following day at 08:30), (2) increased ambient temperature up to 28 °C (temperature controls were adjusted at 08:30, taking approximately 30 min to reach maximum temperature, and settings were returned to 21 °C the following morning at 08:30, taking approximately 15 min to reach set temperature), and (3) footpad pain stimulated by two injections of Freund’s adjuvant containing inactivated *Mycobacterium tuberculosis* suspended in oil (Sigma-Aldrich; St. Louis, MO, US) given 8 h apart, with the cleanest foot per hen being injected with 0.6 mL of adjuvant directly into the footpad. Hens were then monitored for 10 min to ensure no unexpected adverse effects. Injections and monitoring occurred between 09:00–09:30 and at 17:00–17:30. Footpad pain was always the final disruptor applied in order to prevent any carry over effects, as it was unclear exactly how long the adjuvant injection effects would last. The order of periods per day per batch is given in Table 2. Undisturbed periods only occurred in batches 1 and 4; recovery periods always followed disturbed periods. Disturbance treatments were applied for 24 h (08:30–08:30). EEG devices were attached to birds at 09:30 and removed the following day at 08:30 for charging and routine husbandry (e.g., feeding, cleaning drinkers, egg collection), resulting in 23 h of data. At 19:00, birds were manually placed on perches to ensure that video footage of sleep behaviours during the lights-off period was captured. One type of disturbance treatment was used in a single 24 h period, and each bird experienced all three disturbed periods in the study. Following a disturbed period, all disrupters were stopped at 08:30 the following morning. A recovery period always followed disruption, in which birds were recorded using EEG and video cameras with no additional interruptions or alterations to routine husbandry (apart from placing birds on the perches at 19:00). In undisturbed periods, we also used EEG devices and cameras without any of the disruptors, and these periods were used as a means of showing a baseline for sleep behaviour, but only on a night prior to exposure to disruptions (this being the only period that could truly be classed as undisturbed). Devices were removed on the morning of the seventh day following recording, and the study ended. All experimental birds were humanely euthanised via intravenous or intraperitoneal injection of pentobarbital sodium (200 mg/mL, Euthatal; Merial Animal Health Ltd.; Essex, UK) at 1 mL/kg body weight on day 8.

### 2.7. Data Collection and Processing

EEG data were processed as per the protocol outlined in [34]. The first 4 s of every minute of recording was scored as either awake, SWS, REM sleep or artefact, and the proportion of time spent in each state calculated. This totalled 1.5 h of EEG activity per bird per day, 9.2 h per bird, and a total of 92 h analysed across the entire study. Videos of hen behaviour were used to identify specific postures associated with different sleep states, and to confirm unihemispheric states or states that were difficult to define based solely on EEG data, such as between wakefulness and REM sleep (Table 3). Specific behaviours were observed during disruption periods, including drooping wings and panting under increased ambient temperature, and standing on one foot in the case of footpad pain.

### 2.8. Statistical Analysis

Data were grouped into 2 h (during lights-off) or 3.5 h (during lights-on) time intervals in order to account for potential temporal effects. Intervals for birds in which the total proportion of missing data + artefact > 0.25 were omitted from further analysis after visual inspection of distributions. Additionally, with respect to the lights-on period only, due to the final time interval (05:00–08:30) occurring on the following calendar day, there is only a single interval in two batches from which undisturbed periods could be generated; it is for this reason that undisturbed periods are not included in the statistical analysis. The counts for each sleep state were calculated by summing the individual instances of each behaviour for each bird in each interval from which proportions were calculated. Each day starting at 09:30 was classified by ‘period’, as either ‘disturbed’, ‘recovery’ or ‘undisturbed’. Within disturbed periods, the three different disturbances were further split into treatment (feed deprivation, increased ambient temperature, footpad pain) resulting in a five-level factor (i.e., recovery, undisturbed, and the three treatments). In order to normalise residuals, proportions of sleep behaviours were angular transformed prior to fitting linear mixed models (LMMs). LMMs were applied to each sleep state, and for lights-off and lights-on. The fixed effects tested were time interval, period and then treatment adjusted for period, and the interaction between them. The test of treatment after each period in this model is an explicit test of the differences between the three treatments on disturbed nights. The random effects were batch, bird, experimental day (24 levels) and time interval nested within experimental day (120 levels for lights-off, and 84 levels for lights-on). *p* values were based on approximate F tests using the Satterthwaite and Kenward–Roger methods for denominator degrees of freedom. Estimates of means ± standard errors (SEs) were obtained from the models with the fixed effects of time interval, treatment (five levels), and the interaction between them, and were back-transformed onto the proportion scale to aid interpretation. Note that since each sleep state is modelled separately by LMMs, the mean proportions across sleep states will not necessarily sum exactly to one. Mean values are presented with lower and upper bounds calculated through the subtraction of the SE from the mean (lower bound) or addition of the SE to the mean (upper bound), which was subsequently back-transformed, and the least and greatest values identified. Post hoc tests between estimated means were carried out using pair-wise comparisons with Tukey’s HSD test. *p* values were considered significant at ≤0.05. A model was also investigated with six treatment levels (three disturbances and three recovery periods following the application of each disturbance) in order to determine if there were any carry-over effects of the disturbances. As there were no significant effects of the three disturbances within the recovery period, only the models described above with recovery fitted as a single level are reported in the results. Data processing and statistical analyses were carried out in the R system for statistical computing version 4.2.2 [48], which was accessed via RStudio 2022.10.0 Build 353 (RStudio Team, 2022). The additional packages used in the analysis were emmeans, ggplot2, lme4, lmerTest, Matrix, parameters and performance [49,50,51,52,53,54,55].

## 3. Results

The 10 birds completed the trial with no adverse health effects and with the approximate maintenance of their body weight throughout the study (mean body weight change over 94 days: −52.5 ± 85.1 g). The scoring of the different sleep states resulted in a total of 76,286 observations across all birds. After collating data at the level of sleep state and then subdividing the data into lights-off (2 h intervals) and lights-on (3.5 h intervals), there were 1200 observations across all lights-off periods and 828 observations across all lights-on periods. Removing observations for which the proportion of missing + artefact exceeded 0.25 resulted in the exclusion of 172 (14%) observations from the lights-off data and 196 (24%) observations from the lights-on data.

### 3.1. Lights-Off

**Figure 3:**
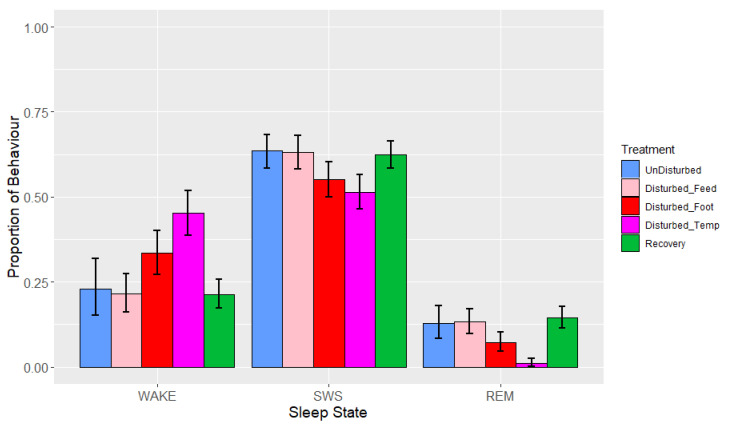
Effects during lights-off by period (undisturbed, disturbed, recovery) and treatments within disturbed periods (‘feed’ = feed deprivation, ‘foot’ = footpad pain, ‘temp’ = increased ambient temperature) on proportions of states (wakefulness (WAKE), slow-wave sleep (SWS) and rapid eye movement (REM) sleep). Values are back-transformed means ± SEs estimated from LMMs fitted to angular transformed proportions, with the fixed effects of time interval, treatment (five levels), and the interaction between them.

During lights-off, the observed mean proportions ± standard deviations (SDs) of sleep behaviours over all nights (after removal of artefact) were 0.28 ± 0.11 (wakefulness), 0.60 ± 0.09 (SWS) and 0.12 ± 0.07 (REM sleep). Resting behaviour was not observed during the lights-off periods. Time interval, period and treatment adjusted for period were significant across all of the sleep states, but there were no significant interactions (Table 4). The means estimated from the LMMs (Figure 3) indicated that birds spent (as a proportion) 0.21–0.45 of the lights-off period awake, 0.51–0.64 in SWS, and 0.01–0.14 in REM sleep over the different periods and treatments.

There was a highly significant effect of time interval during lights-off on all three sleep states (Table 4). Averaging over periods and treatments, post hoc tests showed that wakefulness was highest and SWS was lowest at 03:00–05:00 compared to the other four time intervals (both *p* < 0.01) (Table 5), whereas REM was highest between 23:00–03:00 compared to all other lights-off intervals.

**Figure 4:**
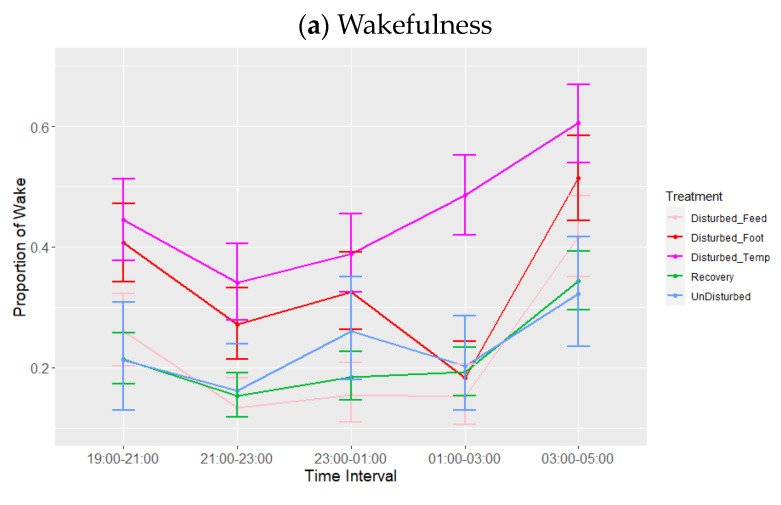

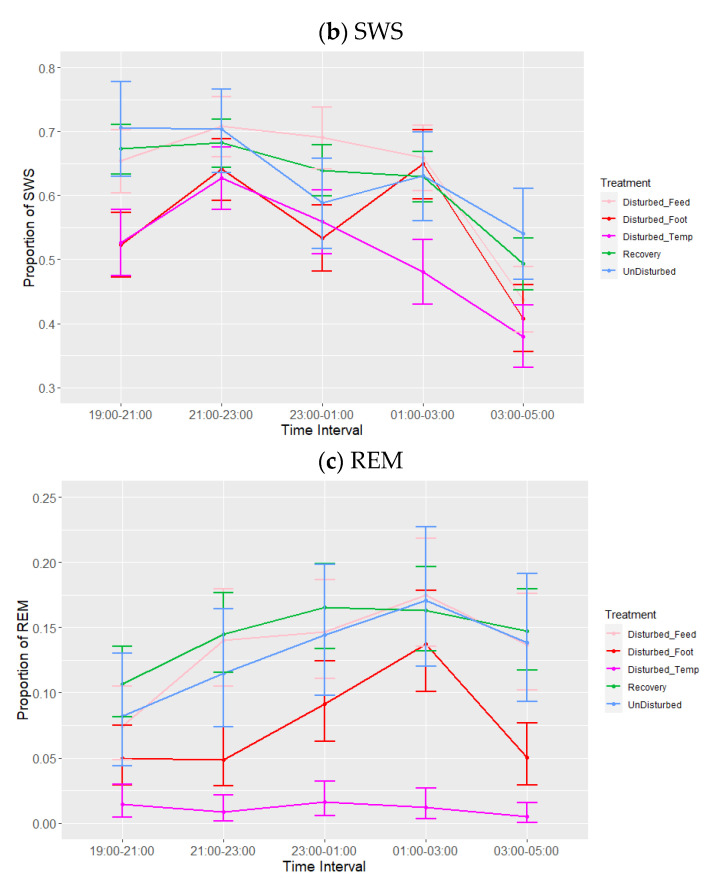
Effects during lights-off of treatment adjusted for period on proportions of states: (**a**) wakefulness (WAKE), (**b**) slow-wave sleep (SWS) and (**c**) rapid eye movement (REM) sleep. Values are back-transformed means ± SEs estimated from LMMs fitted to angular transformed proportions with the fixed effects of time interval, treatment (five levels), and the interaction between them.

There was a significant effect of period and treatment (adjusted for period) on the three sleep states (Table 4, Figure 3). Proportions of wakefulness during lights-off were higher during temperature disturbance compared to feed deprivation (*p* = 0.006), as well as relative to both recovery (*p* < 0.001) and undisturbed periods (*p* = 0.054). No other treatment comparisons differed in their proportion of wakefulness. Similarly, the proportion of SWS was lower during temperature disturbance relative to feed deprivation disturbance (*p* = 0.048), as well as relative to recovery (*p* = 0.026) but not undisturbed periods (*p* = 0.165). SWS did not differ between increased ambient temperature and footpad pain treatments (*p* = 0.863). REM sleep was significantly lower during temperature disturbance, relative to all other periods or treatments (*p* < 0.01). Additionally, footpad pain disturbance resulted in reduced REM sleep compared to recovery periods only. There were no significant effects of interactions on any of the sleep states, although the interaction between time interval and treatment adjusted for period did trend towards significance for REM (*p* = 0.07, Table 4, Figure 4a–c). This trend of marginal interaction for compositional data is the result of the effects of increased ambient temperature on REM sleep such that when it decreased, wakefulness increased. This effect is markedly different for REM sleep compared to the other sleep states, given that REM sleep was so reduced that the normal pattern of increase as the lights-off period progressed was non-existent; this is particularly evident between 01:00 and 03:00. The results of the additional analysis performed using the alternative model with recovery divided into three periods following each type of treatment were not significant during the lights-off period (minimum *p* = 0.94).

### 3.2. Lights-On

**Figure 5:**
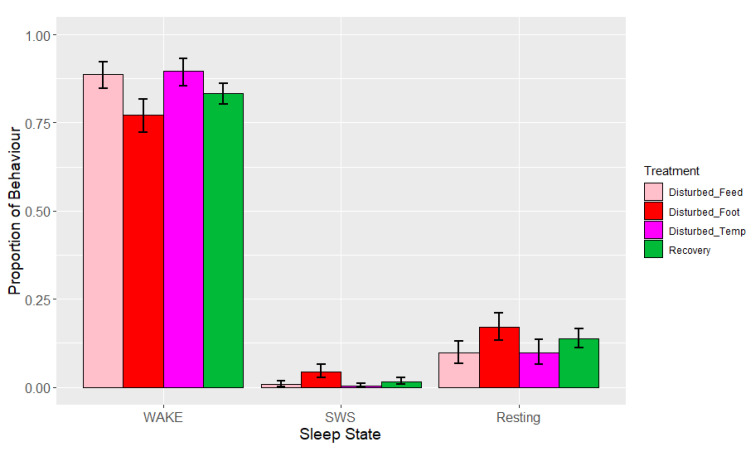
Effects during lights-on by period (disturbed, recovery) and treatment within the disturbed period (‘feed’ = feed deprivation, ‘foot’ = footpad pain, ‘temp’ = increased ambient temperature) on the proportions of states (wakefulness (WAKE), slow-wave sleep (SWS) and resting). Values are back-transformed means ± SEs estimated from LMMs fitted to angular transformed proportions with the fixed effects of time interval, treatment (five levels), and the interaction between them.

The time interval between 09:30 and 12:00 was excluded from analysis, as the 1 h period of device charging and animal husbandry resulted in a >0.25 proportion of missing data/artefacts, leading to automatic exclusion. During lights-on, the observed mean proportions ± SDs of sleep behaviours over all periods were 0.82 ± 0.05 (wakefulness), 0.03 ± 0.02 (SWS) and 0.15 ± 0.05 (resting). REM was not observed during the lights-on period. Time interval and treatment adjusted for period were significant across these states, but the effect of period and the interactions were not significant (Table 6). The means estimated from the LMMs (Figure 5) indicated that birds spent (as a proportion) an average of 0.77–0.90 of the lights-on period awake, 0.01–0.04 in SWS, and 0.10–0.17 resting over the different periods and treatments.

There was a significant effect of time interval on all three states. Additionally, with respect to the lights-on period only, due to the final time interval (05:00–08:30) occurring on the following calendar day, there is only a single interval in two batches from which undisturbed periods could be generated; it is for this reason that undisturbed periods are not included in the statistical analysis. Averaging over the periods and treatments, the post hoc tests showed a trend toward wakefulness being higher (*p* = 0.069) and resting lower (*p* = 0.083) between 15:30 and 19:00, compared to the other two time intervals, whereas SWS was significantly higher at 12:00–15:30 (*p* = 0.010) (Table 7).

**Figure 6:**
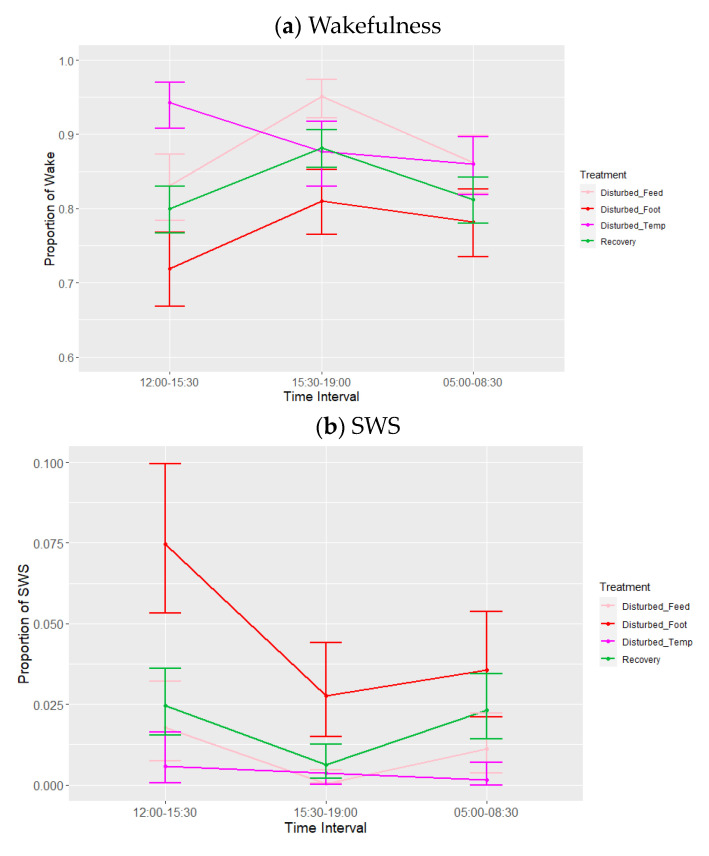

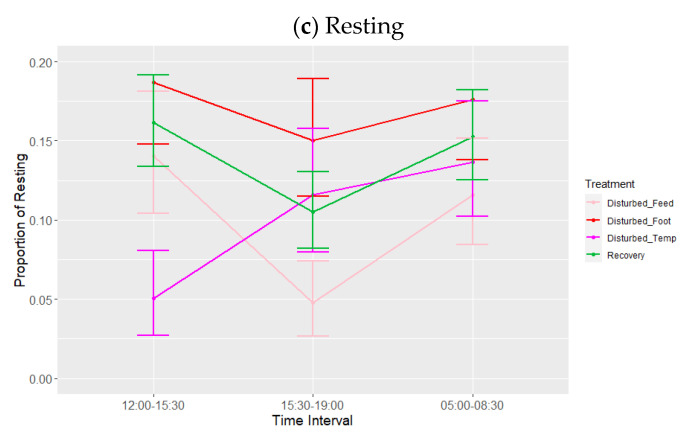
Effects during lights-on of treatment adjusted for period on proportions of states: (**a**) wakefulness (WAKE), (**b**) slow-wave sleep (SWS) and (**c**) resting. Values are back-transformed means ± SEs estimated from LMMs fitted to angular transformed proportions with the fixed effects of time interval, treatment (five levels), and the interaction between them.

There was also a significant effect of treatment (adjusted for period) (Table 6, Figure 5) on lights-on behaviour. Post hoc testing showed that the proportion of time spent in wakefulness was lower, and SWS was higher, during footpad pain disturbance relative to feed deprivation (*p* = 0.008) and increased ambient temperature (*p* < 0.010). The proportion of time spent in SWS was also higher during footpad pain disturbance compared to recovery days (*p* = 0.015). There was a tendency towards less SWS during temperature disturbance relative to recovery periods (*p* = 0.085). Lastly, the proportion of time spent resting was significantly greater with footpad pain compared to feed deprivation only (*p* = 0.040). There were no effects of feed deprivation disturbance on any of the observed behaviours. There were no significant effects of period or any interactions on the sleep states, although the interaction between time interval and treatment adjusted for period did trend towards significance for wakefulness (*p* = 0.071) and resting (*p* = 0.075) (Table 6, Figure 6a–c), for which the trend for increased ambient temperature differed from the other disturbances, with more wakefulness and less resting during 12:00–15:30. Lastly, the results of the additional analysis performed using the alternative model were also not significant during the lights-on period (minimum *p* = 0.10).

## 4. Discussion

### 4.1. Lights-Off

Laying hens followed the sleep trend that has been observed previously across both mammals and birds: specifically a higher proportion of SWS than REM sleep at the beginning of the lights-off period, which steadily decreased with time and was replaced by REM sleep, which was highest towards the end of the lights-off period [27,34,56]. 

During lights-off, hens spent 28% of time in wakefulness, 60% in SWS, and the remaining 12% in REM sleep. This is in keeping with our previous study [34], though it is worth noting that here, there was a 5% reduction in REM sleep and a compensatory increase in wakefulness, most likely as a result of these longer-term disturbances. Other avian sleep studies have found comparable amounts of wakefulness, SWS and REM sleep in animals such as pigeons and non-migratory sparrows [57,58,59]. 

Throughout the lights-off period, wakefulness increased and SWS decreased towards the end of the night. This decrease in SWS as well as a steady increase in REM sleep is typical of the progress of sleep in both mammals and birds [60,61,62,63,64]. The type of period also had a significant effect on the proportion of sleep observed, with wakefulness, SWS and REM sleep all being significantly altered during disturbed periods relative to recovery periods. The lack of significance from the additional alternative model within the lights-off period suggests that either there is no carry-over effect of disturbances on sleep onto the next night, or there is sufficient recovery during the lights-on period immediately following disturbance. These results are generated from the estimates of the LMM, the nature of which do not allow for post hoc testing to pick up pairwise differences as precisely, given the reduced power of the study. Furthermore, REM sleep was significantly lower during disturbed periods compared to both recovery and undisturbed periods. REM sleep appears to be more sensitive to disturbance and deprivation, as it has a lowered threshold to arousal [65]. For example, a study by Aulsebrook et al. [66] looking at the effects of bright artificial light at night in pigeons and magpies found that the presence of bright light significantly reduced REM sleep, while effects on SWS were less consistent. Similar results have been found in cats and rats [67,68]. 

The three disturbances had different effects on sleep, with increased temperature having the greatest effect. Increased ambient temperature resulted in significantly more wakefulness, less SWS compared to undisturbed and recovery periods and feed deprivation disturbance, and significantly lower REM sleep compared to all other periods and disturbance treatments. Connections between both ambient and body temperature and sleep behaviour have been established in other species. For example, Borbely and Tobler [69] observed that ambient temperature plays a strong role in sleep induction. This would suggest that there is a threshold of temperature beyond which sleep is either drastically reduced or altogether eliminated. This is supported by several studies (e.g., [70,71,72]), which found that alterations of ambient temperature tend to affect the timing and periodicity of sleep, while altering temperature far outside the thermal neutral zone can affect sleep duration as well. A study in bats found that when ambient temperatures exceeded 35 °C, no sleep was observed [73]. Additionally, a study by Opp et al. [74], in which gulls were presented with heated copper eggs, found that heating the eggs up to 42 °C significantly reduced sleep behaviour while increasing panting behaviour, and increasing egg temperature to 45 °C eliminated 75% of all sleep. Stuber et al. [75] observed that incremental (5 °C) increases in ambient temperature caused great tits to wake up 30% more frequently, most often towards the beginning of the dark phase. Behavioural postures are also influenced by ambient temperature, with kangaroo rats spending more time in stretched out positions when exposed to increased ambient temperatures [76]. Here, increased temperature almost eliminated REM sleep and reduced SWS on disturbed nights and did not appear to have any carry-over effects on sleep behaviours during recovery nights. With increasing global temperatures, in countries where the ambient temperature is over 21 °C for significant parts of the year, REM sleep may be diminished or altogether eliminated if commercial housing environments are unable to regulate temperatures. Long-term, elevated temperatures may also cause livestock mortality during housing or transport [77,78]. However, while elevated temperatures during transport are relatively brief, increased ambient temperature during housing could last in the order of months.

The findings of this study contradict those of previous studies with regard to pain and hunger. Here, induced footpad pain only reduced REM sleep compared with recovery periods, and food withdrawal (to induce hunger) had no effect on sleep states. In contrast, Borbely [38] found that rats feed deprived for 80 h showed a marked decrease in REM sleep, which returned on the same night upon refeeding. Likewise, Jacobs and McGinty [39] observed that after 6–11 days of feed deprivation in rats, all sleep had disappeared, with REM sleep being eliminated before SWS. It is possible that the lack of feed deprivation effects on sleep was due to 24 h being an insufficient period to have any effect, even though 24 h feed deprivation has been shown to increase the stress hormone plasma corticosterone in broilers; however, direct comparisons are difficult to draw given that broilers are selected for having a greater appetite [79]. Astheimer et al. [80] further proposed that the perception of food deprivation as a stressor is very likely dependent on the body condition of individuals and the availability of energy stores (body fat). As the birds used in this study were adults specifically chosen to be in good physical condition, this may also be a reason as to why feed deprivation had no effect on sleep behaviour. A study by De Jong et al. [81] found that while feed restriction in juvenile broiler breeders increased plasma corticosterone levels and resulted in a change in their activity levels during the day, there was no change to nighttime activity levels between feed restricted and control birds. These findings suggest that while lack of feed induces stress, sleep may be less affected in adult birds with relatively short periods of deprivation.

The limited effects of footpad pain were unexpected. Foot shocks in rats increased alertness by 47%, and significantly reduced total sleep time by 22% [46]. Instances of foot problems and associated pain in hens are dependent on housing (e.g., cages resulting in fewer instances of bumblefoot), but in general are a common occurrence [43,82]. Bumblefoot is a localised and often painful foot lesion that occurs as a result of infection, and is an indicator of poor welfare [44,83,84]. Here, we set out to mimic bumblefoot in a controlled, timed manner by causing an artificial swelling in the foot, which appeared to be sensitive to the touch and thus presumably painful. However, while there were reductions in SWS and REM sleep relative to undisturbed or recovery nights, these reductions were at most intermediate between those of feed deprivation and increased ambient temperature. Anecdotally, birds were observed perching on one foot throughout the night and rousing when the injected foot made contact with the perch. Because of this, it is highly likely that footpad pain increased the degree of sleep fragmentation without necessarily altering the proportion of time spent within the three states. Additionally, as only one foot was injected in order to simulate footpad pain, it is possible that birds may have perched on one foot, allowing them to partially mitigate the disturbance effect and continue to engage in sleep. Due to the nature of the data collection, it is not possible to comment on the level of sleep consolidation experienced by the birds. Future studies on sleep disruption could benefit from treating both feet to increase the discomfort felt during roosting at lights-off.

### 4.2. Lights-On

The proportion of time that laying hens spent in wakefulness, SWS and resting during the lights-on period was consistent with our previous study [34], with most time spent in wakefulness, only 3% in SWS, and the remaining ~15% engaged in resting behaviour. Given that laying hens are a diurnal species, the fact that they spent the majority of time awake during the lights-on period is not surprising [61,85]. Alvino et al. [86] observed that broilers with sufficient levels of light during the day showed a consistent pattern of activity, spending the majority of the lights-on period awake, although studies using EEGs in white leghorns have observed sleep constituting 49% of the day [87]. It is worth noting, however, that the authors attributed a sleep-like state (18% of the observed 24 h behaviour) to this proportion. Without this addition, sleep would constitute 31% of the day, which, even combining SWS and resting together, would far exceed the findings of the present study. The findings during lights-on periods were more similar regardless of period than those of the lights-off periods, which may suggest a lack of carry-over effects from disturbed sleep. This is a surprising finding, given the significant effects of increased ambient temperature on sleep during the lights-off period. The findings of the additional alternative model also not being significant during the lights-on period support the above finding that there were no carry-over effects of the applied disturbances, even immediately following deprivation. Thus, it may be that the disturbances themselves have more of an acute effect, or more likely that the duration of disturbance was not sufficient to elicit a compensatory response. 

While time interval did have a significant effect on the three states observed during the day, this was solely due to a greater proportion of SWS in the early afternoon relative to the later afternoon (just prior to lights-off). This may be evidence for a midday napping period, though given the overall small proportions of sleep observed during the day, this is unlikely. However, Bäckman et al. [88] observed that in migratory birds, there are clear peaks of resting behaviour during the day, while Connelly et al. [62] reported that magpies engage in small bouts of sleep between approximately 10:00 and 13:00, and Johnsson et al. [63] showed that 12 h of sleep deprivation results in a 35% increase in daytime SWS in magpies. It may be that we failed to capture ‘catch-up’ SWS information because of a lack of data in the 09:30–12:00 interval. 

Contrary to what was observed during lights-off, period did not have a significant effect on any of the sleep states, though the disturbance treatment did. While increased ambient temperature had the greatest effects on sleep during lights-off, its effect during lights-on was small. In contrast, footpad pain reduced wakefulness and increased SWS relative to both other disturbances. SWS was also increased with footpad pain in comparison with recovery periods. Birds also spent a greater proportion of time resting when experiencing footpad pain compared to other disturbances. It may be that simulated bumblefoot swelling was painful enough to reduce overall activity (e.g., reduced foraging, fewer trips to the feeder/drinker); previous studies have shown that sickness and disease result in greater amounts of sleep and resting (as reviewed in [89]). As with lights-off, these effects may have been more pronounced if both feet had been treated. While single-foot swelling may have small effects on nighttime sleep, it has other implications for bird welfare if birds subsequently reduce other desirable behaviours such as eating, drinking, foraging, etc.

## 5. Conclusions

The above findings highlight the sensitivity of sleep to specific disturbances. Extending the timing and duration of disturbances resulted in more significant effects on sleep behaviour than seen in our previous study [34], despite an unclear level of carry over effects into days following the cessation of disturbance. The lack of effect of 24 h feed deprivation (and to a lesser extent, simulated bumblefoot in a single foot) suggests that these disturbances may not necessarily be of immediate concern in relation to sleep behaviour, and therefore welfare, at least in comparison to heat stress. It may be that birds were able to mitigate the effects of footpad pain during the night, resulting in a lack of statistically significant differences compared to undisturbed or recovery periods. It is possible that further extending the duration or severity of these treatments may magnify their effects on sleep behaviour in laying hens to a significant degree. The effects of increased ambient temperature, however, are especially noteworthy, given increasing global temperatures as a direct result of climate change. It is likely that these trends will continue, introducing an additional welfare concern for commercially kept poultry. Future poultry housing units will need to contain air-conditioning systems in order to combat heat stress, which affects sleep, and to maintain welfare standards.

## Figures and Tables

**Table 1:** Experimental and companion bird allocation according to their ID numbers and batch. Experimental birds E5 and E6 were culled prior to the start of this study due to health concerns.

Batch	Companion Bird ID Numbers	Experimental Bird ID Numbers
1	C1, C2	E1, E2, E3
2	C3, C4	E4,
3	C3, C4	E7, E8, E9
4	C5, C6	E10, E11, E12

**Table 2:** Experiment diary for batches 1–4. Study 1 refers to Putyora et al. [34]. Birds experienced a different type of period per day (recorded over 23 h): undisturbed, disturbed, or recovery. Disturbances were applied at 09:30 on a disturbed recording day and continued until 08:30 the following day. There were three disturbance treatments, and each bird experienced each treatment once.

Day	Batch 1 and 4	Batch 2 and 3
Day −21	Surgery	Surgery
Day −20–0	Study 1	Study 1
Day 1	Undisturbed sleep recording	Disturbed sleep recording (feed deprivation)
Day 2	Disturbed sleep recording (increased ambient temperature)	Recovery sleep recording
Day 3	Recovery sleep recording	Disturbed sleep recording (increased ambient temperature)
Day 4	Disturbed sleep recording (feed deprivation)	Recovery sleep recording
Day 5	Recovery sleep recording	Disturbed sleep recording (footpad pain)
Day 6	Disturbed sleep recording (footpad pain)	Recovery sleep recording
Day 7	Equipment switched off	Equipment switched off

**Table 3:** Ethogram of sleep behaviour assessment criteria and corresponding electroencephalograph (EEG) patterns.

Video	EEG	Definition
Awake	Waking EEG (low-amplitude high-frequency waves)	Bird is clearly awake and engaged in activity, including walking, preening, nest building, laying, feeding, drinking, foraging, panting and standing on one leg.
Resting	Waking EEG (low-amplitude high-frequency waves)	Minor and infrequent head movements while in a stereotypic sleep posture (sitting with wings folded or head retracted into the breast), and not engaged in any active behaviours. One or both eyes may be closed with occasional opening. EEG is the same as awake EEG.
Sleep	Slow-wave sleep (SWS) EEG (high-amplitude low-frequency waves)	Bird is in a stereotypic sleep posture (sitting or perching with wings folded and head retracted) with one or both eyes closed. EEG has transitioned from waking/resting to SWS.
Sleep	Rapid eye movement (REM) sleep EEG (low-amplitude high-frequency waves—must be preceded by SWS)	Bird is in a stereotypic REM sleep posture (sitting or perching with wings relaxed and head hanging downwards) with both eyes closed. EEG has transitioned from SWS to REM sleep.

**Table 4:** F tests for the effects of time interval (‘Time’, time of day divided into 2 h blocks), period (disturbed, recovery, undisturbed), treatment (feed deprivation, increased ambient temperature, footpad pain) adjusted for period and their interactions (Time × Period and Time × Treatment) on wakefulness, slow-wave sleep (SWS) and rapid eye movement (REM) sleep during lights-off periods from LMMs. F values, with numerator and denominator degrees of freedom (ndf, ddf, respectively), are shown.

	Wakefulness					SWS					REM				
	Time	Period	Treatment	Time × Period	Time × Treatment	Time	Period	Treatment	Time × Period	Time × Treatment	Time	Period	Treatment	Time × Period	Time × Treatment
ndf,ddf	4,210	2,19	2,17	8,209	8,209	4,212	2,18	2,16	8,211	8,211	4,209	2,17	2,15	8,208	8,208
F value	23.2	7.6	9.2	0.6	1.6	28.8	4.1	5.3	1.0	1.5	7.2	16.7	20.5	0.4	1.8
*p* value	<0.001	0.004	0.002	0.768	0.127	<0.001	0.035	0.017	0.475	0.177	<0.001	<0.001	<0.001	0.915	0.070

**Table 5:** Effects of time interval during lights-off on proportions of states (wakefulness, slow-wave sleep (SWS) and rapid eye movement sleep (REM)). Values are back-transformed means, with back-transformed mean—SE and back-transformed mean + SE forming the lower and upper bounds, respectively, estimated from LMMs fitted to angular transformed proportions with the fixed effects of time interval, treatment (five levels), and the interaction between them. Where superscripts (a,b) are different within a column, means are significantly different (*p* < 0.05) (obtained through post hoc Tukey’s HSD tests).

	Wakefulness			SWS			REM		
	Proportion	Lower Bound	Upper Bound	Proportion	Lower Bound	Upper Bound	Proportion	Lower Bound	Upper Bound
19:00–21:00	0.26 ^b^	0.22	0.31	0.65 ^a^	0.61	0.69	0.08 ^b^	0.05	0.10
21:00–23:00	0.18 ^b^	0.15	0.22	0.68 ^a^	0.64	0.72	0.10 ^b^	0.08	0.13
23:00–01:00	0.24 ^b^	0.20	0.28	0.61 ^a^	0.57	0.64	0.12 ^a^	0.10	0.15
01:00–03:00	0.22 ^b^	0.18	0.26	0.62 ^a^	0.58	0.66	0.14 ^a^	0.11	0.17
03:00–05:00	0.39 ^a^	0.35	0.44	0.48 ^b^	0.44	0.52	0.11 ^b^	0.08	0.13

**Table 6:** F tests for effects of time interval (‘Time’, time of day divided into 3.5 h blocks), period (disturbed, recovery), treatment (feed deprivation, increased ambient temperature and footpad pain) adjusted for period, and their interactions (Time × Period and Time × Treatment) on wakefulness, slow-wave sleep (SWS) and resting during lights-on from LMMs. F values, with numerator and denominator degrees of freedom (ndf, ddf, respectively), are shown.

	Wakefulness					SWS					Resting				
	Time	Period	Treatment	Time × Period	Time × Treatment	Time	Period	Treatment	Time × Period	Time × Treatment	Time	Period	Treatment	Time × Period	Time × Treatment
ndf,ddf	2,42	1,42	2,42	2,42	4,42	2,31	1,42	2,38	2,27	4,38	2,39	1,40	2,39	2,40	4,40
F value	5.7	0.8	9.6	0.1	2.3	7.1	0.1	13.7	0.6	0.7	3.9	0.9	5.5	0.3	2.3
*p* value	0.007	0.385	<0.001	0.929	0.071	0.003	0.786	<0.001	0.540	0.586	0.028	0.336	0.008	0.777	0.075

**Table 7:** Effects during lights-on of time interval on proportions of states (wakefulness, slow-wave sleep (SWS) and resting). Values are back-transformed means, with back-transformed mean—SE and back-transformed mean + SE forming the lower and upper bounds, respectively, estimated from LMMs fitted to angular transformed proportions with the fixed effects of time interval, treatment (five levels), and the interaction between them. Where superscripts (a,b) are different within a column, means are significantly different (*p* < 0.05) (obtained through post hoc Tukey’s HSD tests).

	Wakefulness			SWS			Resting		
	Proportion	Lower Bound	Upper Bound	Proportion	Lower Bound	Upper Bound	Proportion	Lower Bound	Upper Bound
12:00–15:30	0.83 ^a^	0.81	0.86	0.03 ^b^	0.02	0.04	0.13 ^a^	0.11	0.15
15:30–19:00	0.89 ^a^	0.86	0.91	0.01 ^a^	0.00	0.01	0.10 ^a^	0.08	0.14
05:00–08:30	0.83 ^a^	0.81	0.85	0.01 ^a^	0.01	0.02	0.14 ^a^	0.12	0.17

## Data Availability

The datasets collected, generated and analysed for this study are available from the corresponding author via Zenodo repository (8195533).
